# mCRPC Patients Receiving ^225^Ac-PSMA-617 Therapy in the Post–Androgen Deprivation Therapy Setting: Response to Treatment and Survival Analysis

**DOI:** 10.2967/jnumed.121.263618

**Published:** 2022-10

**Authors:** Mike Sathekge, Frank Bruchertseifer, Mariza Vorster, Ismaheel O. Lawal, Otto Knoesen, Johncy Mahapane, Cindy Davis, Amanda Mdlophane, Alex Maes, Kgomotso Mokoala, Kgomotso Mathabe, Christophe Van, de Wiele, Alfred Morgenstern

**Affiliations:** 1 Department of Nuclear Medicine; University of Pretoria and Steve Biko Academic Hospital, Pretoria, South Africa;; 2 Nuclear Medicine Research Infrastructure, Pretoria, South Africa;; 3 European Commission, Joint Research Centre, Karlsruhe, Germany;; 4 Nuclear Technology Products, Pelindaba, South Africa;; 5 Katholieke University Leuven, Kortrijk, Belgium;; 6 Department of Urology; University of Pretoria and Steve Biko Academic Hospital, Pretoria, South Africa; and; 7 Ghent University, Ghent, Belgium

**Keywords:** ^225^Ac-PSMA, ADT, therapy response, PSA response, prostate carcinoma

## Abstract

^225^Ac-PSMA-617, targeting the prostate-specific membrane antigen (PSMA), which is overexpressed on prostate cancer cells, has shown a remarkable therapeutic efficacy in heavily pretreated patients with metastatic castration-resistant prostate carcinoma (mCRPC). Here, we report on treatment outcome and survival using this novel treatment modality in a series of 53 patients with mCRPC directly after their androgen deprivation treatment (ADT). **Methods:** ^225^Ac-PSMA-617 was administered to 53 such patients. ^68^Ga-PSMA PET/CT was obtained at baseline, before every treatment cycle, and on follow-up to select patients for treatment, determine the activity to be administered, and assess their response. Serial prostate-specific antigen (PSA) measurements were obtained for response assessment. **Results:** The median age of the patients was 63.4 y (range, 45–83 y). In total, 167 cycles were administered (median, 3; range, 1–7). Forty-eight patients (91%) had a PSA decline of at least 50%, and 51 patients (96%) had any decline in PSA. ^68^Ga-PSMA PET findings became negative in 30 patients. In the multivariate analysis, a PSA decline of at least 50% proved predictive of both progression-free survival (PFS) and overall survival (OS), and platelet count also proved predictive for PFS. The median estimated OS was 9 mo for patients with a PSA decline of less than 50% but was not yet reached at the latest follow-up (55 mo) for patients with a PSA decline of 50% or more. The estimated median PFS was 22 mo for patients with a PSA decline of at least 50% and 4 mo for patients with a PSA decline of less than 50%. No severe hematotoxicity was noted, and only 3 patients had grade III–IV nephrotoxicity. The commonest toxicity seen was grade I–II xerostomia, observed in 81% of patients. **Conclusion:** In 91% of 53 patients with mCRPC, treatment with ^225^Ac-PSMA-617 immediately after ADT resulted in at least a 50% decrease in PSA level. Furthermore, a PSA decline of at least 50% proved the single most important factor predicting PFS and OS after ^225^Ac-PSMA-617 treatment. Of interest, median OS in patients with a PSA decline of at least 50% was not yet reached at the latest follow-up (55 mo). These favorable results suggest that it would be of major clinical relevance to perform a prospective randomized study comparing ^225^Ac-PSMA-617 with current standard-of-care treatment options such as enzalutamide, abiraterone acetate, and docetaxel after ADT.

## Visual Abstract

**Figure:**
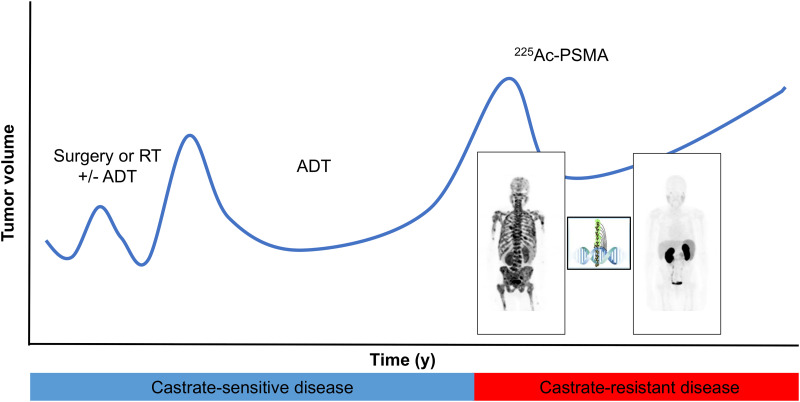

Prostate cancer is the second most frequent malignancy (after lung cancer) in men worldwide, accounting for approximately 4% of all deaths caused by cancer in men (*1**,**2*). Although the 5-y survival rate of localized prostate carcinoma is nearly 100%, the 5-y survival rate for patients with metastatic castration-resistant prostate carcinoma (mCRPC) is only about 30% (*3*). Standard-of-care treatment for mCRPC is androgen deprivation therapy (ADT), which normalizes serum levels of prostate-specific antigen (PSA) and produces an objective tumor response in over 90% of patients. However, despite an initial favorable response to ADT, most patients with mCRPC eventually experience disease progression within an average of 18–36 mo after treatment initiation (*4**,**5*). Once ADT-resistant or castration-resistant, mCRPC patients are treated with other options such as abiraterone acetate, enzalutamide, chemotherapy, ^223^Ra-dichloride, or sipuleucel-T, the choice of which depends substantially on patient preference, current symptoms, burden of disease, and local availability (*5*).

Treatment of prostate carcinoma patients in low- to middle-income countries is challenging. Because of the lack of regular PSA screening, most prostate carcinoma patients in low- to middle-income countries present with metastatic disease at initial diagnosis (*6*). In addition, because of fear of associated side effects, some patients often refuse both ADT and chemotherapy. Furthermore, abiraterone and enzalutamide are not easily accessible to most patients.

^225^Ac-PSMA-617, targeting the prostate-specific membrane antigen (PSMA), which is overexpressed on prostate cancer cells, has shown remarkable therapeutic efficacy in heavily pretreated mCRPC patients (*7**–**11*). When ^225^Ac-PSMA-617 therapy is applied in dose-deescalation fashion, the most prevalent treatment-related toxicity is grade 1–2 xerostomia, making the therapy an acceptable alternative for mCRPC patients in low- to middle-income countries who refuse chemotherapy because of fear of associated side effects or to whom novel treatment options such as enzalutamide or abiraterone are not readily available.

We previously reported on the favorable outcome and toxicity results from ^225^Ac-PSMA-617 therapy in a small group (17 patients) with mCRPC (*12*). In this study, we report treatment outcome and survival in a larger series (53 patients) directly after ADT.

## MATERIALS AND METHODS

This was a retrospective review of patients with histologically confirmed mCRPC treated with ^225^Ac-PSMA-617 radioligand therapy. In patients who presented with early-stage disease, primary therapy was by radical prostatectomy, external-beam radiotherapy to the prostate gland, or brachytherapy. In patients who presented with metastatic disease, initial therapy was by ADT using surgical or medical castration. Eligibility for ^225^Ac-PSMA-617 radioligand therapy was based on PSA progression: a minimum of 2 rising PSA values from a baseline measurement, with the measurements separated from one another by at least 1 wk. Inclusion criteria included mCRPC precluding treatment with localized therapy such as radiotherapy, patient refusal of chemotherapy, and lack of access to second-generation antiandrogen therapy such as abiraterone and enzalutamide (i.e., patients without medical insurance). Exclusion criteria included urinary tract obstruction and bone marrow suppression (Common Terminology Criteria for Adverse Events grade 3 or more). The decision to treat patients with ^225^Ac-PSMA-617 was made in a multidisciplinary setting in our hospital for patients who eventually experienced disease progression despite an initial favorable response to ADT within an average of 18 mo after treatment initiation. At the time of treatment, all patients were aware that ^225^Ac-PSMA-617 had not yet received regulatory approval for use in the routine care of patients with mCRPC. They understood that the treatment was applied on a compassionate basis for patients who refused available life-prolonging treatment options or had no access to these novel therapies. All patients therefore provided written informed consent to undergo treatment with ^225^Ac-PSMA-617 with a full awareness of its possible complications, including xerostomia, bone marrow suppression, renal impairment, and potential currently unknown side effects. The institutional review board (Research Ethics Committee of the Faculty of Health Sciences, University of Pretoria, reference number 173/2021) approved this retrospective study, and the requirement to obtain informed consent was waived.

### Patient Preparation

The patients first underwent ^68^Ga-PSMA-11 PET/CT imaging and were deemed suitable candidates for therapy with ^225^Ac-PSMA-617 if uptake by the tumor lesions was higher than physiologic uptake in the normal liver parenchyma. A full blood count, liver function tests, and measurements of electrolytes, urea, and creatinine were performed before treatment commencement and were repeated 2 wk before subsequent treatment cycles to determine patient fitness for them. ^68^Ga-PSMA-11 PET/CT was repeated after each subsequent treatment cycle to determine the burden of residual tumor to guide dose deescalation.

### Preparation and Administration of ^225^Ac-PSMA-617

PSMA-617 was radiolabeled with ^225^Ac as described previously (*8**,**12*). The initial administered activity of ^225^Ac-PSMA-617 was 8 MBq for all patients. For subsequent treatment cycles, the administered activity was deescalated to 7, 6, or 4 MBq on the basis of the response to earlier treatments as assessed on repeat ^68^Ga-PSMA-11 PET/CT as previously described (*12*). Treatments were repeated every 8 wk, provided that a continued response was demonstrated, no limiting toxicity developed, and residual tumor demonstrating ^68^Ga-PSMA-11 avidity was shown on PET/CT imaging.

### Safety

All patients were observed for a minimum of 4 h after ^225^Ac-PSMA-617 administration to detect any immediate side effects. Within 2 wk before the first cycle of treatment, a baseline assessment was performed of hemoglobin level, leukocyte count, platelet count, glomerular filtration rate, and liver function. Except when the clinical situation warranted more frequent follow-up, these blood tests were repeated 2 wk before subsequent cycles of treatment (i.e., every 8 wk). After completion of all treatment cycles, these blood tests were repeated every 12 wk until disease progression or death. Patients who developed toxicity were followed up until resolution or death. In addition to undergoing the blood tests, patients reported any observed side effects during treatment or on follow-up. When the patients came for each cycle of treatment or follow-up, they were asked about side effects known to occur with PSMA-based radioligand therapy. Toxicity was defined according to the Common Terminology Criteria for Adverse Events, version 5.0.

### Treatment Response Evaluation

Treatment response was evaluated using serial measurements of serum PSA values and ^68^Ga-PSMA-11 PET/CT imaging. ^68^Ga-PSMA-11 PET/CT was repeated every 8 wk (before each treatment cycle) and subsequently every 12 wk after treatment completion until disease progression or death. PSA response was defined as a PSA decline of at least 50% of the baseline value, according to the Prostate Cancer Working Group 3 criteria. Follow-up ^68^Ga-PSMA-11 PET/CT was used to evaluate the status of initially identified metastatic lesions fulfilling the inclusion criteria on the baseline PET/CT scan. We used PSMA PET/CT criteria to categorize patients as responders or nonresponders. Favorable responders were categorized as showing stable disease, a partial response, or a complete response on PSMA PET/CT imaging; nonresponders were patients with progressive disease on PSMA PET/CT (*13*). The PSMA response was defined as complete if all lesions with tracer uptake disappeared; as partial if uptake lessened and tumor volume decreased by more than 30% on PET; as stable if there was a change in uptake and no more than a 30% change in tumor volume on PET, without evidence of new lesions; and as progressive if at least 2 new lesions appeared, uptake increased, or tumor volume increased by at least 30% on PET (*13**–**15*).

### Statistical Analysis

Statistical analysis was performed using SPSS, version 28.0 (IBM). The Kolmogorov–Smirnov test was used to check whether data were normally distributed. Quantitative variables were compared using a paired Student *t* test and ANOVA when normally distributed or using a Mann–Whitney test and Kruskal–Wallis test when not normally distributed.

For univariate and regression analysis, we dichotomized values according to the median values in cases of continuous variables. We also dichotomized the following clinical covariates: Gleason score; number of treatment cycles; the presence of bone, visceral, and lymph node metastases; PSA response (≤50% reduction or >50% reduction); undetectable PSA levels; and normalization of the ^68^Ga-PSMA-11 PET findings. Progression-free survival (PFS) and overall survival (OS) were estimated by the Kaplan–Meier method and log rank testing to examine the predictive value of dichotomized variables and other clinical risk factors for disease control and OS. Multivariate analysis was performed using Cox regression and included, in sequential order of statistical significance, variables that were found to be significant in the univariate analysis followed by the interactive terms.

Finally, the χ^2^ test was used to determine differences in proportion when appropriate.

## RESULTS

### Patient Characteristics

Patient characteristics are shown in Table 1. Fifty-three mCRPC patients were included. Their median age was 63.4 y (range, 45–83 y). Twenty-three had an Eastern Cooperative Oncology Group score of 0, 19 had a score of 1, and 11 had a score of 2. Six patients had isolated lymph node involvement (stage IVA disease); the remaining patients all had bone metastases (stage IVB disease), and 6 of these patients also had visceral metastases (1 patient with both brain and liver metastases, 4 patients with liver metastases, and 1 patient with lung metastases). The median PSA level before treatment was 466 ng/mL (range, 102–4,405 ng/mL). The mean hemoglobin level was 11.5 g/dL (range, 6.1–16 g/dL), the median platelet count was 293,000/μL (range, 48,000–762,000/μL), the mean white blood cell count was 7,090/μL (range, 3,100–14,870/μL), and the median alkaline phosphatase level was 188 IU/L (range, 82–1796 IU/L).

**TABLE 1:** Patient Characteristics

Characteristic	Value
No. of patients included	53
Median age (y)	63.4
Eastern Cooperative Oncology Group score of 0 or 1 (*n*)	42
Eastern Cooperative Oncology Group score of 2 (*n*)	11
Median PSA level (ng/mL)	466
Median alkaline phosphatase level (IU/L)	188
Median hemoglobin value (g/dL)	11.5
Bone metastases (*n*)	47
Lymph node metastases (*n*)	36
Visceral metastases (*n*)	6
Lung	1
Liver	5
Brain	1
Local therapy to prostate (*n*)	
Prostatectomy	31
Radiotherapy	11
No local therapy	11

In total, 167 cycles were administered (median, 3; range, 1–7). Seven patients received 1 cycle; 15 patients, 2 cycles; 11 patients, 3 cycles; 11 patients, 4 cycles; 2 patients, 5 cycles; 6 patients, 6 cycles; and 1 patient, 7 cycles. Eight patients continued with hormonal treatment despite progressive disease under these agents; their urologist or oncologist did not want to stop these medications because clinical benefit was still assumed.

### Safety

Administration of ^225^Ac-PSMA-617 was well tolerated. The commonest toxicity seen was grade I–II dry mouth, observed in 81% of patients. No patient with grade III dry mouth was seen, and no patient discontinued treatment because of this side effect. No patient with grade IV bone marrow toxicity was seen. Anemia was the most common manifestation of hematotoxicity, seen in 15% of patients (7 patients with grade I–II anemia and 1 patient with grade III). Any grade of renal failure was seen in 19% of patients (7 with grade I–II, 2 with grade III, and 1 with grade IV). Details on toxicity in the treated patients are in Table 2.

**TABLE 2:** Toxicity Profiles of 53 Patients Treated with ^225^Ac-PSMA-617

Characteristic	Grade I–II	Grade III	Grade IV
Xerostomia	43 (81%)	0	0
Anemia	7 (13%)	1 (2%)	0
Leukopenia	4 (7%)	1 (2%)	0
Thrombocytopenia	5 (9%)	0	0
Renal failure	7 (13%)	2 (4%)	1 (2%)

### Response to ^225^Ac-PSMA-617 Therapy

**FIGURE 1.**
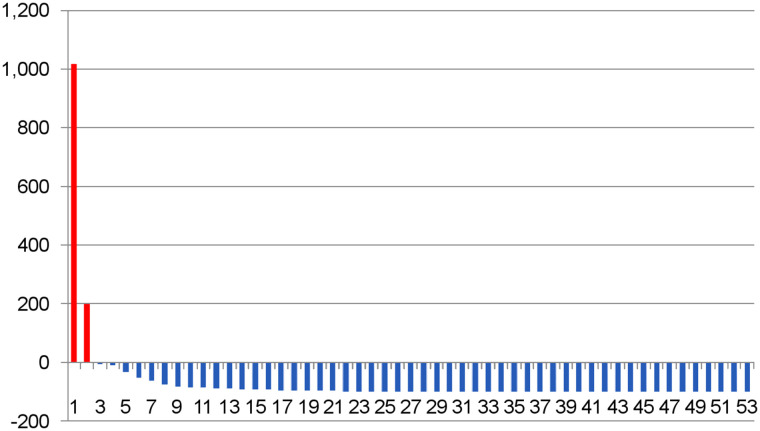
Waterfall plot demonstrating percentage change in PSA levels after treatment with ^225^Ac-PSMA-617 in patient cohort (*x*-axis = number of patients; *y*-axis = percentage change).

After ^225^Ac-PSMA-617 treatment, 48 patients (91%) had a PSA decline of at least 50%, and 51 patients (96%) had any decline in PSA (Fig. 1). PSA became undetectable in 19 patients (36%). ^68^Ga-PSMA PET images became negative in 30 patients (57%); that is, avidity was similar to background blood-pool activity in all prostate cancer lesions after treatment with ^225^Ac-PSMA-617.

### OS

**FIGURE 2.**
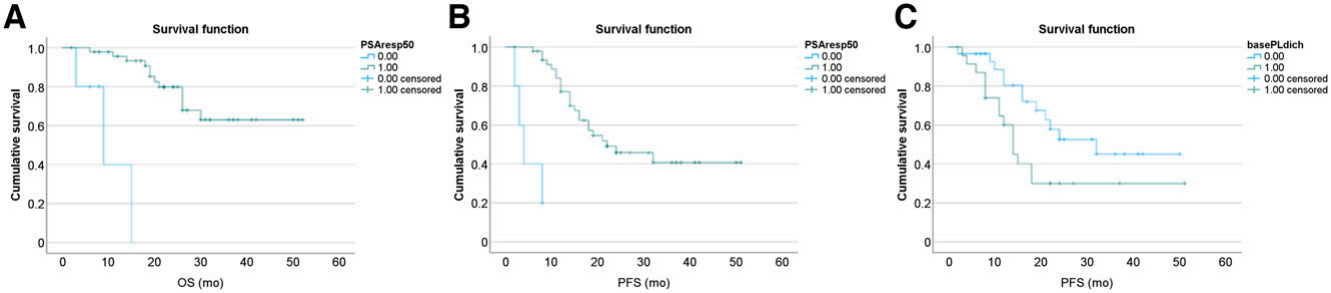
Kaplan–Meier curves of PSA-based OS of the entire cohort (PSA decline of ≥ 50% [green curve] and percentage of PSA decline < 50% [blue curve]) (A), PFS of the entire cohort (PSA decline of ≥ 50% [green curve] and percentage of PSA decline < 50% [blue curve]) (B), and PFS stratified by platelet counts (platelet counts < 293,000/mL [blue curve] and platelet counts > 293,000 [green curve]) (C).

At the time of data analysis, 15 patients (28%) had died, and all deaths seemed directly related to their underlying mCRPC. In the univariate analysis, only a PSA decline of at least 50% proved significantly associated with a favorable OS (*P* < 0.001) (Table 3). When included in the multivariate analysis with age and Gleason score as covariates, the level of statistical significance was retained (*P* < 0.001). The median estimated OS was 9 mo for patients with a PSA decline of less than 50% but was not yet reached at the latest follow-up (55 mo) for patients with a PSA decline of 50% or more (Fig. 2). The OS of patients with stage III disease (lymph node involvement only, 6 patients) did not significantly differ from that of patients with stage IV disease (the remaining 47 patients) (*P* = 0.186).

**TABLE 3:** Univariate Analysis of Relationship Between Studied Variables and Survival

Variable	PFS	OS
Age	0.180	0.748
Eastern Cooperative Oncology Group score	0.077	0.772
Gleason score	0.596	0.774
Previous local radiotherapy	0.304	0.916
Baseline PSA level	0.972	0.888
PSA ≥ 50% decline	<0.001*	<0.001*
PSA undetectable	0.014*	0.132
Visceral metastases	0.937	0.772
Lymph node involvement	0.289	0.942
Bone metastases	0.459	0.186
No. of treatment cycles	0.650	0.097
ALP	0.727	0.886
Hemoglobin	0.090	0.132
Platelet count	0.041*	0.602
White blood cell count	0.373	0.605
Radiologic response	0.006*	0.407
PSMA-negative	0.026*	0.418

* *P* < 0.05.

### PFS

During follow-up, 27 patients (51%) showed disease progression. In univariate analysis, the following parameters proved significantly related to PFS: a PSA decline of at least 50% (*P* < 0.0001), undetectable PSA (*P* = 0.014), platelet count (*P* = 0.041), ^68^Ga-PSMA-11 PET/CT–based response (*P* = 0.006), and negative findings on ^68^Ga-PSMA PET (*P* = 0.026), (Table 3; Fig. 2). When included in the multivariate analysis, only a PSA decline of at least 50% (*P* = 0.002) and platelet count (*P* = 0.047) retained statistical significance. The estimated median PFS for patients with a PSA decline of at least 50% was 22 mo, whereas that for patients with a PSA decline of less than 50% was 4 mo. The number of patients who relapsed but had negative PSMA PET findings after treatment (7 of 23) did not significantly differ from the number who relapsed but did not have negative PSMA PET findings after treatment (19/30) (*P* = 0.027).

## DISCUSSION

Various early preclinical and clinical studies have provided a rationale for combining ADT with radiotherapy for the management of localized prostate cancer (*16*). For instance, in nude mice bearing Shionogi adenocarcinoma allografts, Zietman et al. demonstrated that ADT reduced the dose of radiotherapy necessary to control 50% of the tumor and that the timing of ADT is important for achieving this effect; orchidectomy was significantly more effective if performed 12 d before radiotherapy than if performed during or after radiotherapy (*17**,**18*). In Dunning prostate cancer–bearing rats, temporary ADT for 14 d before radiotherapy resulted in a significant lengthening of tumor growth (*19*). Furthermore, ADT was shown to downregulate vascular expression of growth factor, causing apoptosis of endothelial cells and normalization of tumor vascularization, thereby increasing oxygenation (*20*). Also, in a series of 237 prostate carcinoma patients, Milosevic et al. identified a broad heterogeneity in prostate cancer oxygenation, a prerequisite for radiotherapy efficacy, with the median partial pressure of O_2_ ranging from 0 to 75 mm Hg (*20*). On the basis of these studies, various randomized phase III trials have been conducted that showed a significant clinical benefit from adding ADT to radiotherapy when treating intermediate-risk primary prostate carcinoma (*21*). As opposed to the beneficial radiotherapy-enhancing effects of short-term ADT administered in combination with radiotherapy for primary intermediate prostate carcinoma, long-term ADT administration in the metastatic setting resulting in androgen independence is often characterized by a remarkable resistance to treatment options that trigger apoptosis via the caspase cascade, including radiotherapy. Various factors responsible for radiation resistance in androgen-independent prostate carcinoma have been implicated, including increased levels of interleukin-6, neuroendocrine differentiation, Ack-1 androgen receptor phosphorylation, the existence of intrinsic cancer stem cells, and epithelial–mesenchymal transition, among others (*22**–**25*).

In the series presented, 91% of mCRPC patients had at least a 50% reduction in their initial PSA value after ^225^Ac-PSMA-617 treatment, and neither baseline PSA nor alkaline phosphatase level significantly differed between responders and nonresponders. This percentage of patients exceeds by far those found in early clinical studies demonstrating at least a 50% PSA reduction: approximately 10% of patients treated with ipilimumab, sunitinib, cabozantinib, or ^223^Ra-dichloride (Xofigo; Bayer) and approximately 30%, 40%, and 50% of patients treated with abiraterone, cabazitaxel, or enzalutamide (*26*). Our findings suggest that radiation resistance to α-emitting agents after ADT is not a significant issue, as is the case with, for instance, β-emitting agents in some patients. In this regard, reported response rates of mCRPC patients to ^177^Lu-PSMA-617 have varied from 10.6% to 69% (*27*). Although these response rates were obtained in heterogeneous patient cohorts receiving various other treatments after ADT, lower response rates to ^177^Lu-PSMA-617 as opposed to ^225^Ac-PSMA-617 may be anticipated given the low linear-energy-transfer value of 0.7 keV/μm for ^177^Lu when compared with 100 keV/μm for ^225^Ac (*28*). The high linear-energy-transfer value of ^225^Ac results in a high level of radiobiologic effectiveness when compared with β-radiation, requiring fewer particle tracks to induce cell death via induction of predominant DNA-strand breaks, among others, obviating cellular oxygen to induce its therapeutic effect (*27**,**29*). Although Xofigo is also an α-therapy targeting bone lesions only, it will most likely have less success than ^225^Ac-PSMA-617, which targets both soft-tissue and bone lesions. This likelihood is also supported by recent information that both nodal and visceral metastases have been underestimated and understudied in patients with advanced prostate cancer and the fact that patients with visceral metastases invariably have a poorer prognosis than patients with bone-only metastases (*30*).

In our series, hemoglobin levels, a surrogate marker for molecular oxygen level, proved unrelated to treatment outcome. Furthermore, part of the high PSA response observed in our series may be due to the abscopal effect, attributed to irradiation-induced immune mechanisms such as exposure to tumor antigen, increased maturation of antigen-presenting cells taking up antigen released by dying cells, and production of interleukin-6 and tumor necrosis factor-α, as well as changes in the tumor microenvironment for improved recruitment of effector T cells (*31*). In this regard, Gorin et al., evaluating an α-emitting ^231^Bi-labeled antibody for tumor cell irradiation of mouse xenografts, found that the treatment induced a protective antitumor effect by induction of tumor-specific T cells against a secondary tumor cell injection (*32*). Furthermore, Czernin et al. showed, in a mouse model of mCRPC, that a combination of ^225^Ac-PSMA-617 and an inhibitor of programmed cell death protein 1 achieved better tumor control than monotherapy with either agent alone (*33*). In our series, 2 patients proved unresponsive to ^225^Ac-PSMA-617 treatment despite high uptake of the ligand on the baseline scan. As shown by Kratochwil et al., in such patients mutations in DNA-damage-repair and checkpoint genes are frequently found. Future studies assessing the role of DNA-damage-repair–targeting agents in combination with ^225^Ac-PSMA-617 therapy in overcoming radiation resistance in these patients are of interest (*34*).

A PSA decline of at least 50% to assess efficacy of treatment, as recommend by the Prostate Cancer Working Group 3, proved the single most important factor predicting PFS and OS after ^225^Ac-PSMA-617 treatment in our patient cohort. The importance of PSA decline was also demonstrated in our previous study (*35*). The median estimated PFS was 4 mo for nonresponders and 22 mo for responders. Although median OS in the nonresponding group was 9 mo, median OS had not yet been reached at the last follow-up (55 mo) (Fig. 2). A first chemotherapy-naïve patient exceeding 5 y of complete remission after ^225^Ac-PSMA-TAT was reported in Germany (*36*). Overall, our OS data in this small cohort suggest that ^225^Ac-PSMA-617 has efficacy superior to that of chemotherapy (enzalutamide, abiraterone acetate, or docetaxel) administered in a comparable setting (post-ADT mCRPC patients having received no other treatment targeting their mCRPC) (*28**–**32*). Regarding the use of docetaxel in the mCRPC setting, 2 large phase 3 randomized, controlled trials published in 2004 (the TAX327 and SWOG9916 trials) found a median OS of 18.9 mo versus 16.5 mo and of 17.5 mo versus 15.6 mo for the control group receiving mitoxantrone, considered the standard of care at that moment (*37**,**38*). In the COU-AA-302 trial, comparing abiraterone acetate (1,000 mg once daily) plus prednisone or placebo plus prednisone in 1,048 patients randomly assigned to receive either of these treatment options, median OS for patients in the abiraterone acetate group was 34.7 mo (*39*). With regard to enzalutamide, in the double-blind phase 3 PREVAIL trial, in which 1,717 patients were randomly assigned to receive either enzalutamide at a dose of 160 mg or placebo once daily, the median estimated OS for the enzalutamide-treated group was 32.4 mo, versus 30.2 mo for the placebo group (median duration of follow-up for survival, ∼22 mo) (*40*). Recently, the TheraP trial demonstrated that ^177^Lu-PSMA-617, compared with cabazitaxel, in men with mCRPC led to a higher PSA response and fewer grade 3 or 4 adverse events. ^177^Lu-PSMA-617 is a new, effective class of therapy and a potential alternative to cabazitaxel (*41*). Although not directly comparable between earlier-stage and later-stage application of ^225^Ac-PSMA-617 or ^177^Lu-PSMA-617, a couple of studies have shown a better response from PSMA radioligand therapy with ^177^Lu-PSMA-617 in chemotherapy-naïve patients (*42*). Furthermore, our group reported a unique cohort of chemotherapy-naïve men with mCRPC who had upfront treatment with ^225^Ac-PSMA-617 (*12*) and demonstrated a remarkable 88% serum PSA decline by 50% or more after a median of 3 cycles of ^225^Ac-PSMA-617, as is corroborated by this current series. This result is in contrast to an average of 65.4% serum PSA decline by 50% of patients in studies that had a later-stage application of ^225^Ac-PSMA-617 or ^177^Lu-PSMA-617 (*8**–**18**,* *30*). Although this remarkable response is exciting and holds much promise for ^225^Ac-PSMA-617 in treating men with mCRPC, it may also represent a response achieved in less aggressive disease (*42*). mCRPC evolves, acquiring more aggressive behavior as different lines of treatment are applied to it. Additionally, of the patients presenting with liver metastasis, none presented with negative PSMA PET results after treatment, nor did any respond favorably to the treatment, as liver metastasis was also negatively correlated with OS in other studies (*30*). Thus, randomized controlled trials will be needed to stratify patients to either ^177^Lu-PSMA-617 or ^225^Ac-PSMA-617 so that the better therapy is administered at the moment in the treatment sequence when it is likely to have the best impact.

Finally, in our study, a high platelet count also proved significantly negatively related to PFS. Although the role of platelets is to stem blood loss after vascular injury, available data suggest that platelets may also interact with tumor cells and endothelial cells, enabling metastases and thereby worsening the prognosis of cancer patients (*43**–**45*). More specifically, platelets were shown to play a role in shielding tumor cells from immune elimination, in promoting arrest and extravasation of tumor cells, and in protecting cancer cells from undergoing apoptosis. Furthermore, experimental data suggest that thrombocytosis is induced by tumor-derived growth factors. Finally, a high pretreatment baseline platelet count has been previously associated with a poor prognosis in patients with ovarian, breast, lung, renal, colorectal, and pancreatic carcinoma (*46*).

In terms of the safety profile, xerostomia remains an adverse effect of concern, and most of our patients experienced dry mouth—commonly after the first cycle of treatment. To reduce the incidence and severity of treatment-induced xerostomia, we practiced the treatment deescalation strategy. Administered activity was reduced to 6 or 4 MBq in subsequent treatment cycles according to the volume of residual tumor load. This strategy is based on the principle of the tumor sink effect, in which more radioligand is available for binding in normal organs when tumor bulk is reduced by successful treatment (*47*). As we reported previously, we believe that this strategy is partially successful because none of our patients has experienced grade III xerostomia or discontinued ^225^Ac-PSMA-617 therapy because of dry mouth (*12**,**35*). Although anemia was also a relatively common toxicity (15%), no other grade 3 or 4 hematotoxicities were noted. Notably, only 3 patients experienced grade III–IV renal function impairment. All 3 of these patients presented with suboptimal renal function before the ^225^Ac-PSMA-617 therapy. This finding warrants medium- to long-term monitoring of renal function of patients treated with ^225^Ac-PSMA-617.

This study was retrospective and consequently bears all the disadvantages of such types of studies, including—in this specific setting—lack of a control group. However, the favorable results suggest that it would be of major clinical relevance to perform a prospective randomized study comparing ^225^Ac-PSMA-617 with standard-of-care treatment options such as enzalutamide, abiraterone acetate, and docetaxel after ADT.

## CONCLUSION

In 91% of this series of 53 mCRPC patients receiving ^225^Ac-PSMA-617 therapy subsequent to ADT, the PSA level decreased by more than 50%. A PSA decline of at least 50% proved the single most important factor predicting PFS and OS after ^225^Ac-PSMA-617 treatment. The median estimated PFS was 4 mo for nonresponders and 22 mo for responders, and the median OS was 9 mo in the nonresponding group and had not yet been reached at the last follow-up (55 mo) in the responding group. ^225^Ac-PSMA-617 is a highly promising option for therapy of mCRPC directly after ADT and warrants further study in randomized trials.

## DISCLOSURE

No potential conflict of interest relevant to this article was reported.

## Notes

KEY POINTS **QUESTION:** What is the efficacy of ^225^Ac-PSMA-617 in the post-ADT setting in men with mCRPC? **PERTINENT FINDINGS:** ^225^Ac-PSMA-617 administered in the post-ADT setting to men with mCRPC resulted in a PSA response in 91% of patients and produced an undetectable level of serum PSA in 36%. A decline in serum PSA by at least 50% was significantly associated with a longer OS. A PSA decline of at least 50%, a low pretreatment platelet level, and radiographic response on ^68^Ga-PSMA-11 PET/CT were significant predictors of a longer PFS. **IMPLICATIONS FOR PATIENT CARE:** ^225^Ac-PSMA-617 is a viable treatment option that may be considered in men who develop mCRPC after ADT, especially if approved treatment options are not available or are contraindicated.
